# Low prevalence of Borna disease virus 1 (BoDV-1) IgG antibodies in humans from areas endemic for animal Borna disease of Southern Germany

**DOI:** 10.1038/s41598-019-56839-4

**Published:** 2019-12-27

**Authors:** Dennis Tappe, Christina Frank, Ruth Offergeld, Christiane Wagner-Wiening, Klaus Stark, Dennis Rubbenstroth, Sebastian Giese, Erik Lattwein, Martin Schwemmle, Martin Beer, Jonas Schmidt-Chanasit, Hendrik Wilking

**Affiliations:** 1 Bernhard-Nocht-Institut für Tropenmedizin, Hamburg, Germany; 2 Robert Koch-Institut, Berlin, Germany; 3 State Health Department Baden-Wurttemberg, Stuttgart, Germany; 4 Friedrich-Loeffler-Institut, Greifswald/Insel Riems, Germany; 5 Institut für Virologie, Universitätsklinikum Freiburg, Freiburg, Germany; 6 Euroimmun Medizinische Diagnostika, Lübeck, Germany; 7 Faculty of Mathematics, Informatics and Natural Sciences, University of Hamburg, Hamburg, Germany

**Keywords:** Viral epidemiology, Risk factors

## Abstract

Borna disease virus-1 (BoDV-1) was recently discovered as cause of severe and often fatal encephalitis in humans. BoDV-1 is known to cause neurological disease in horses and sheep mainly in South and Central Germany. The virus is maintained in bicolored white-toothed shrews (*Crocidura leucodon*). The incidence of infection and risk factors in humans are completely unresolved. Veterinarians may be disproportionally BoDV-1-exposed through contact to animals not recognized to be BoDV-1 infected. We conducted three serosurveys predominantly in endemic areas of South Germany for the presence of BoDV-1-reactive antibodies. Anonymized residual samples from two serosurveys of veterinarians (n = 736) with interview data on exposures and one serosurvey among blood donors (n = 373) were screened with an indirect immunofluorescence antibody test, followed by a newly developed immunoblot as confirmatory assay. One serum from a 55–59-year-old veterinarian who worked in an animal practice and as a meat inspector but none from blood donors tested positive by the screening and confirmatory assays. We show that seropositive individuals are rare even in areas with highest zoonotic risk and in a group with potentially elevated exposure risk. In light of the low seroprevalence demonstrated here, the high case-fatality rate in clinically observed human BoDV-1 infections is even more impressive.

## Introduction

**Figure 1:**
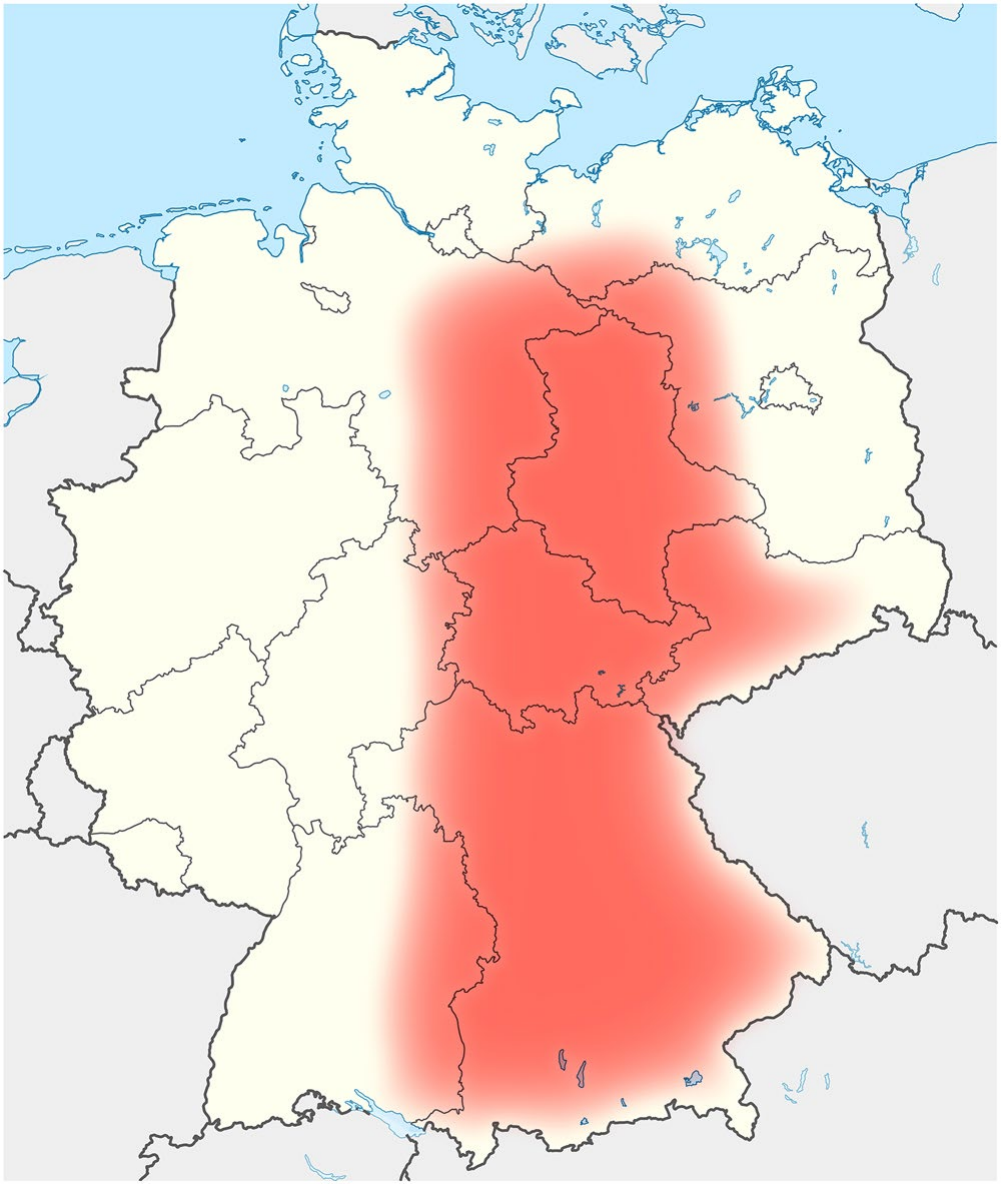
Endemic areas for BoDV-1-infections in Germany. The areas are proposed based on knowledge of geographical distribution of Borna disease in animals or presence of BoDV-1 in shrews. Endemic areas were drafted according to data from reference ^5^.

Borna disease virus 1 (BoDV-1, species *Mammalian 1 orthobornavirus*) was discovered to cause severe human encephalitis in 2018^1,2^. The virus was responsible for two fatal encephalitis cases and one severe but non-fatal brain infection in three recipients of solid organ transplants from the same donor^2^. The donor died without reported neurological disease. Furthermore, in the same year three more patients with fatal BoDV-1 encephalitis – epidemiologically unrelated to each other and to the organ transplant cluster – were detected in Bavaria, Germany^1,3^. The bicolored white-toothed shrew (*Crocidura leucodon*) constitutes the natural reservoir of the virus^4–6^. A broad range of mammals is susceptible to BoDV-1-infections leading to neurological disease^7^. Regions endemic for Borna disease (BD) in spill-over hosts, mainly horses and sheep, in Germany extend from the North of the federal German states of Saxony-Anhalt via Central Thuringia to Bavaria and neighboring districts of Baden-Wurttemberg (Fig. 1). Bavaria is especially affected in geographical extent and incidence of animal BD. Adjacent parts of Switzerland, Liechtenstein, and Austria are also endemic for BD^4,5,8^. Risk factors and the transmission routes from shrews to humans are unknown. One hypothesis is that pet animals or livestock might act as intermediate hosts occasionally bringing people in contact with the virus. While occupational exposure of farmers and horse owners around affected livestock has not been recognized in humans despite decades of familiarity with BD in endemic areas, an exposure to the virus by sporadically infected pets such as cats is likely harder to notice. The rationale for this study is based on the theory that veterinarians who have professional contact to a large number of animals in areas endemic for BD are disproportionally intense exposed to BoDV-1 (population at risk). An increased risk in this specific group could translate into a risk for other groups with intense animal contacts, such as pet owners.

Here, we report the results of multipanel IgG serosurveys for BoDV-1 conducted to obtain first estimates of the frequency of this zoonotic infection in humans, particularly in Bavaria.

## Methods

Panel A and B consisted of anonymized residual serum samples from serosurveys of veterinarians with interview data on illnesses and exposures to animal species^9^. The panel A survey was conducted 2009 at the Bavarian Veterinary Association conference in Rosenheim with 424 participants (364 from Bavaria). This survey was originally conducted as a *Coxiella burnetii* seroprevalence study. Participants recalled high prevalence of exposure to different animal species and to animal bites and needle stick injuries^9^. The panel B survey was conducted in 2010 at the German Veterinary Association conference in Nuremberg, with 312 participants (98 from Bavaria). Panel B was included to cover also areas non-endemic for BoDV-1 in animals. Participants of panel A and B were included after obtaining written informed consent. A standardized questionnaire was issued and serum samples were taken. Data has been anonymized after publishing the *Coxiella burnetii-*estimates and for the purpose of this study the residual samples were used. Studies were approved by the ethical review board of the Charité medical school of Berlin. All methods were performed in accordance with this approval. Panel A and B participants’ median age was 42 (18–74) years, 461 (63%) were female. Panel C was included for comparison and consisted of anonymized sera from 373 healthy blood donors from Bavaria donating blood in October 2018 (21–60-year-old adults in four equally weighted age groups and from the whole state of Bavaria); 170 (46%) of the donors were female.

Testing for anti-BoDV-1 IgG was conducted with an indirect immunofluorescence antibody test (IFAT) using a persistently BoDV-1 infected cell line for screening and uninfected cells of the same cell line as controls^2,10,11^. Positive results were confirmed using an immunoblot with recombinantly expressed BoDV-1 and variegated squirrel bornavirus 1 (VSBV-1) N and P proteins^11^. Sera from confirmed human BoDV-1 encephalitis cases were used as positive controls^2^. A pooled serum of 20 healthy blood donors was used as negative control for both the IFAT and the immunoblot. All sera with intranuclear IFAT patterns indicative of bornavirus infections in dilutions ≥1:10 were considered positive. End-point titers are indicated as the reciprocal dilution of the highest positive dilution factor. Sera that tested positive were treated with increasing concentrations of urea and were again analyzed by IFAT and immunoblot for avidity measurements^12^. Serological testing was performed in a blinded fashion in four different diagnostic centers experienced in bornavirus serology and read by at least 2 specifically trained persons each.

Prevalence and binomial confidence intervals for proportions were calculated with Stata 15.1.

## Results

**Figure 2:**
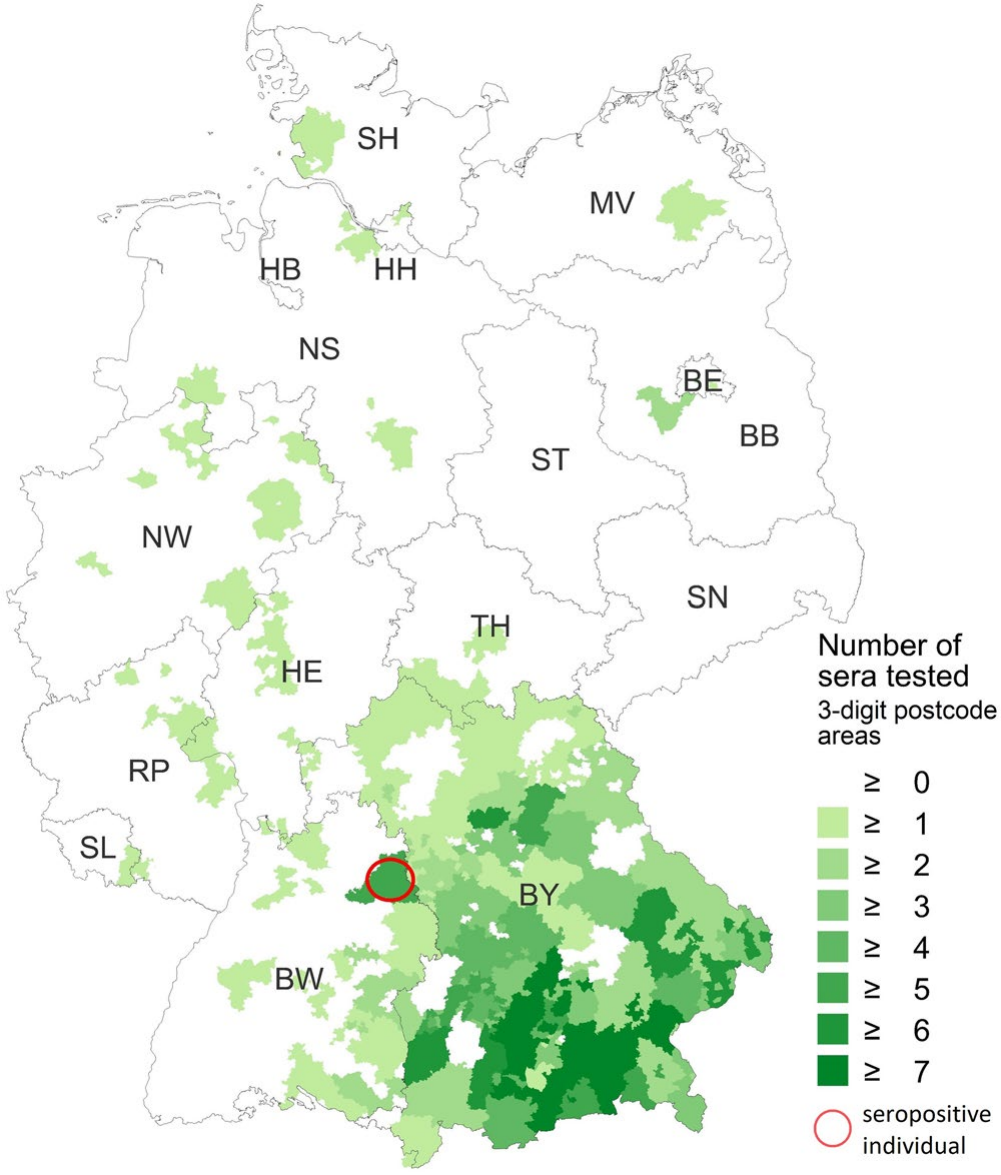
Spatial distribution of residence of veterinarians in a serosurvey for BoDV-1, Germany. Self-reported place of residence by study participants (n = 424) conducted at a conference by the Bavarian Veterinary Association 2009 in Rosenheim (study panel A). The residential area of the seropositive individual is marked with a red circle.

**Figure 3:**
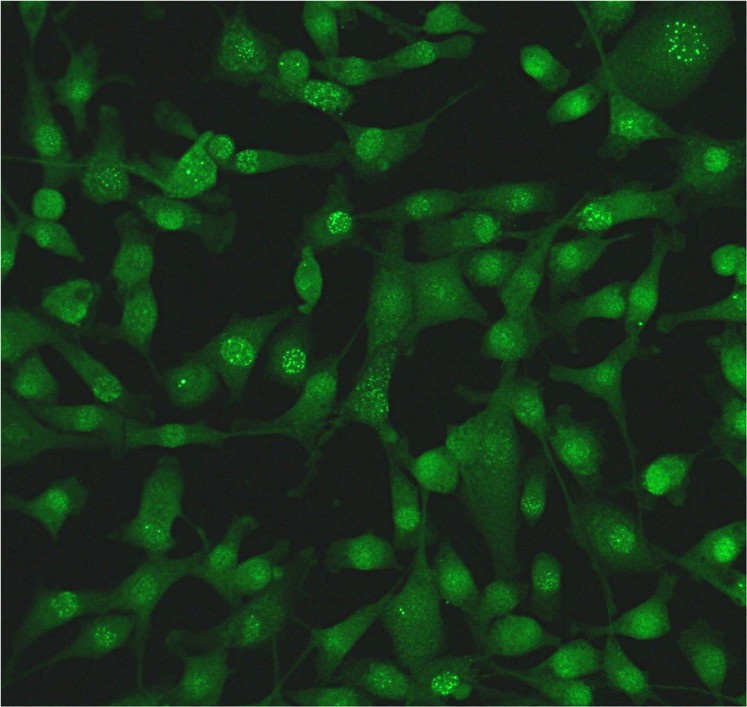
Positive BoDV-1 immunofluorescence antibody test of a serum sample from a veterinarian. Intranuclear indirect immunofluorescence signal typical for BoDV-1 reactive IgG-antibodies using the veterinarian’s serum on a persistently BoDV-1 infected cell line (original magnification x100).

**Figure 4:**
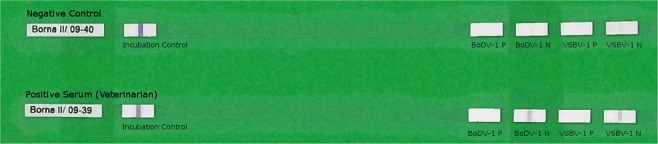
Positive BoDV-1 immunoblot result of a serum sample from a veterinarian. The same serum as shown in Fig. 3 exhibits positive reactions to BoDV-1 N and VSBV-1 N proteins on an IgG-immunoblot with recombinant antigens.

Among the 736 veterinarians (panel A+B), one anti-BoDV-1 IgG positive serum was identified by all four different diagnostic centers (seroprevalence of 0.14% [95%-CI: 0.003–0.75%]). This single positive serum originated from a 55–59-year-old female veterinarian of panel A (seroprevalence of 0.24% [95%-CI: 0.006–1.30%]) from Baden-Wurttemberg near the border with Bavaria (Fig. 2) and exhibited an IFAT IgG titer of 2,560 (Fig. 3). In the immunoblot, it reacted strongly with BoDV-1 N protein (90 arbitrary units; cut-off, 16 arbitrary units), and with lower intensity with VSBV-1 N protein (60 arbitrary units). Reactions against BoDV-1 and VSBV-1 P proteins were negative (1 and 2 arbitrary units, respectively; Fig. 4). BoDV-1-reactive antibodies in the serum showed high avidity, providing unaltered IFAT titers and immunoblot results in the presence of up to 8 M urea. The woman had been working as a veterinarian in a small animal practice and as a meat inspector in a slaughterhouse for 25 years. She had experienced several needle prick injuries and animal bites. She listed suffering from joint pain for five years as health complaint.

Among the 373 blood donors (panel C), no sample tested positive for anti-BoDV-1 IgG (seroprevalence of 0% with an upper confidence bound of 0.98%).

## Discussion

BoDV-1 has long been known for causing severe neurological infections with high fatality rates in accidental animal hosts, particularly in horses and sheep. A large spectrum of mammals is susceptible to natural and experimental infection^7,13–15^. Human BoDV-1 infection is likely confined to areas where clinical BD in farm animals indicates the presence of infected reservoir animals. Infected bicolored white-toothed shrews show no signs of illness but excrete the virus in urine, saliva, and other excretions, and also by skin scaling^5,16^. While it is assumed that grazing animals take up the virus through mucous membranes into underlying nervous tissue^17^, the routes of human infections are unclear. It cannot be excluded that working on a farm or contact to pets (such as cats preying on shrews), might increase the risk for direct or indirect contact with the reservoir animals.

After the detection of BoDV-1 in human patients with severe encephalitis^1,2^ it became apparent that there is an as yet not quantified disease risk. In our study, we found a very low prevalence of BoDV-1-reactive antibodies among veterinarians. Given the sample size, a seroprevalence above 1.3% (upper confidence interval) can be excluded with high probability. The overall risk of infection is apparently very low, even in geographical localities with the highest possibility of exposure (panel A+C) and in population groups with a potentially elevated possibility of exposure (panel A+B). Due to this low seropositivity our study was not able to determine and calculate risk factors for disease. The low seroprevalence in population groups at risk (which reflects rare infection events) in combination with the high case-fatality rate (CFR) of known human BoDV-1-disease suggests an extremely high probability of clinically apparent disease after infection (high clinical manifestation index).

This study has several limitations: As the sera of the veterinarians who had taken part in the study were anonymized, virological, serological or clinical follow-up was not possible for the seropositive woman. No further history of possible neurological episodes could be asked. Sampling of veterinarians and blood donors was performed at different time points so the median birth years of veterinarians are 10 years before the blood donors, thus hampering comparisons between seroprevalence estimates. This study analyzed the current places of residencies of participants and not previous places of residencies, work and animal contacts. Since samples for direct BoDV-1 detection were not available from the seropositive veterinarian, confirmation of a BoDV-1 infection was not possible. Hence, it remains unknown whether the detected BoDV-1-reactive antibodies were actually induced by BoDV-1 or a different orthobornavirus, such as VSBV-1 that was discovered in a cluster of fatal encephalitis cases among exotic squirrel breeders in 2015^10^. Serological assays for bornavirus infection show cross-reactivity within the genus *orthobornavirus*^11,18^. We cannot exclude that the veterinarian had contact to exotic squirrel species, a reservoir of VSBV-1, or animal reservoirs of other, so far unknown, zoonotic orthobornaviruses. However, as the VSBV-1 N reactivity in the immunoblot was lower than the BoDV-1 N reactivity, a VSBV-1 infection is rather unlikely. The absence of reactivity against BoDV-1 P in the immunoblot was not completely unexpected, as it was shown in experimentally infected rats and naturally infected horses that such a constellation may occur^12^. However, in these animals, most often serum antibody reactivities against both BoDV-1 N and P were seen^12^. Finally, we cannot exclude that the serological bornavirus-reactivity in the veterinarian may result from antibodies detecting individual antigenetic epitopes unrelated to, or without any previous contact with, orthobornaviruses^12^.

Moreover, if the clinical manifestation index (those ill among infected) of human BoDV-1-infection and the CFR were actually very high as assumed here, any serosurvey in healthy persons would suffer from a significant healthy participant bias unless they are accidentally surveyed when still in the incubation period. This healthy participant bias would lead to an underestimation of the true prevalence of infection and thus indirectly also of the true incidence of disease.

So far, the donor from the organ transplant-associated BoDV-1 encephalitis cluster^2^ is the only serologically positive person (IFAT titer of 40) known to date without reported neurological disease, but with high evidence of a real BoDV-1 infection as his organs were responsible for subsequent BoDV-1 infections in the recipients. Unfortunately the time of infection could not be estimated in the donor and it is probable that he died during the incubation time of the disease. In previous studies by others^12,19^, seropositive individuals without neurological signs were also detected but a proof of infection by direct BoDV-1 detection was lacking – similar to the seropositive veterinarian described in our investigation. In contrast however, the veterinarian from our study lives in a BoDV-1-endemic area and due to her occupation, an exposition can be plausibly assumed. Further patient groups, such as psychiatric patients^20,21^, were shown to have antibodies reactive to BoDV-1 antigens in low concentrations by various tests. These test results, however, were obviously unrelated to known BD-endemic areas and exposure risk, and likely reflect cross-reactivity or unspecificity^22^ of the assays used.

## Conclusion

Our study revealed one individual with BoDV-1-reactive IgG antibodies among veterinarians, a group with an assumed increased risk of exposure to BoDV-1. This low seroprevalence provides evidence against widespread human BoDV-1 infection which is in contrast to previous work by others^23^. The paucity of seropositive individuals strengthens the notion of human BoDV-1 infections being rare, even in areas with known animal BD. In our study, we applied well-defined human positive control sera which were not available before.

Our study results also show that serosurveys targeting populations at risk or the general population require extraordinary sample sizes to obtain significant results. Therefore, in the future, active case finding among encephalitis patients including subsequent systematic exposure assessment and case-control studies may be more qualified for epidemiological studies on risk factors than serosurveys. On the basis of the current knowledge, healthcare providers should consider testing patients with severe neurological disease of unknown etiology for BoDV-1, especially those who reside in, or traveled to, areas endemic for animal BD.
